# Brain Temperature: Physiology and Pathophysiology after Brain Injury

**DOI:** 10.1155/2012/989487

**Published:** 2012-12-26

**Authors:** Ségolène Mrozek, Fanny Vardon, Thomas Geeraerts

**Affiliations:** Department of Anesthesia and Critical Care, University Hospital of Toulouse, University Paul Sabatier, Toulouse, France

## Abstract

The regulation of brain temperature is largely dependent on the metabolic activity of brain tissue and remains complex. In intensive care clinical practice, the continuous monitoring of core temperature in patients with brain injury is currently highly recommended. After major brain injury, brain temperature is often higher than and can vary independently of systemic temperature. It has been shown that in cases of brain injury, the brain is extremely sensitive and vulnerable to small variations in temperature. The prevention of fever has been proposed as a therapeutic tool to limit neuronal injury. However, temperature control after traumatic brain injury, subarachnoid hemorrhage, or stroke can be challenging. Furthermore, fever may also have beneficial effects, especially in cases involving infections. While therapeutic hypothermia has shown beneficial effects in animal models, its use is still debated in clinical practice. This paper aims to describe the physiology and pathophysiology of changes in brain temperature after brain injury and to study the effects of controlling brain temperature after such injury.

## 1. Introduction

Many popular figures of speech connect brain activity with temperature. It is now well known that, while brain temperature is largely dependent on the metabolic activity of brain tissue, the regulation of these two parameters is complex. The relationship between temperature and metabolism is always interactive. While brain cell metabolism is a major determinant of brain temperature, minor changes in brain temperature can result in significant changes in neural cell metabolism and therefore in brain function. Tight control of brain temperature is critical for optimal brain function under different physiological conditions such as intense physical activity or complete rest.

In intensive care clinical practice, continuous monitoring of core temperature in patients with brain injury is highly recommended [1]. It has been shown that, in cases of trauma, the brain is extremely sensitive and vulnerable to small temperature variations. Indeed, fever is considered a secondary injury to the brain in neurosurgical patients with severe traumatic brain injury [2], subarachnoid hemorrhage [3], or stroke [4], in whom hyperthermia is a frequent phenomenon. In these cases, guided, directed normothermia can be used to limit secondary brain injury. This paper aims to describe the physiology and pathophysiology associated with changes in brain temperature, with particular focus on acutely ill patients suffering from severe traumatic brain injury, stroke, or subarachnoid hemorrhage.

## 2. Physiology of Brain Temperature

**Figure 1:**
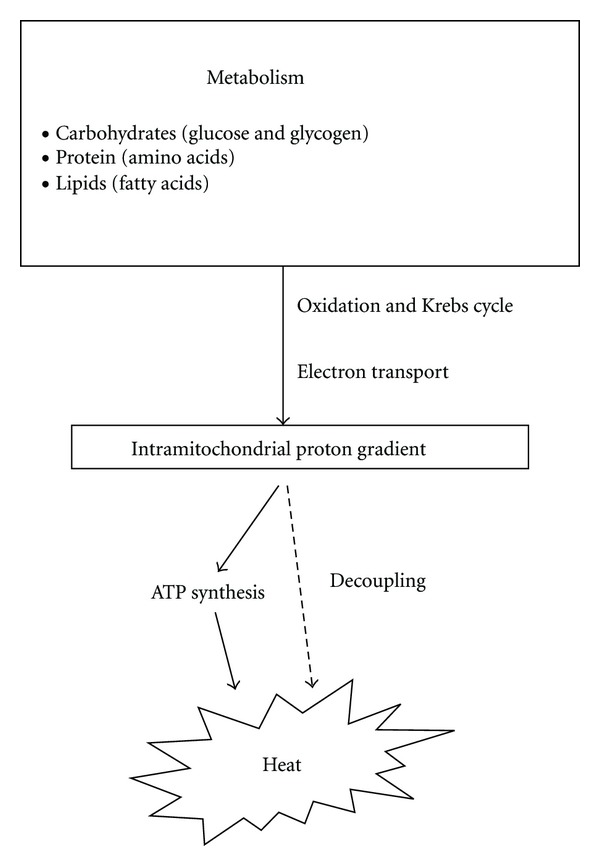
Heat production during energy metabolism. This schema is valid whatever the cell type.

Energy production in humans derives from glucose, protein, and fat metabolism. The end products of aerobic metabolism are carbon dioxide (CO_2_) and water. The production of adenosine triphosphate (ATP), the main intracellular energy storage molecule, is accompanied by heat (Figure 1). The energy lost during electron transport and oxidative phosphorylation is largely converted into heat and contributes to maintaining body temperature at 37°C. The combustion of glucose and protein produces 4.1 kcal/kg, while fat combustion yields 9.3 kcal/kg. Heat production depends, therefore, on energy metabolism [5].

Although the brain represents only from 2 to 3% of human body weight, it uses 20% and 25% of the body's total consumption of oxygen and glucose, respectively. Even at rest, the metabolic activity of brain tissue is high. Energy metabolism in the brain is mainly aerobic; 95% of the glucose used by the brain undergoes oxidative metabolism. Approximately 40% of the energy provided by glucose is used to produce ATP; the remainder (approximately 60%) is converted into heat [5]. Under normal conditions, production of heat within the brain is balanced by its dissipation. In contrast to other organs such as muscles, the heat produced within the brain is not easily dispersed due to the protection of the brain by the skull. Brain temperature depends primarily on three factors: local production of heat, temperature of the blood vessels, and cerebral blood flow. Dissipation of generated heat is improved by vascular anatomical specializations that permit heat exchange.

### 2.1. Heat Exchangers

Heat exchangers vary across species. In felids, arterial blood for the brain flows through a vascular network at the base of the skull. In these species, the carotid artery is very close to the cavernous or pterygoid sinus, which receives cool blood from the mucosal surfaces of the nose. This heat exchange produces selective brain cooling (SBC) that depends on sympathetic activity [6]. In canids, the carotid rete is rudimentary [7]. However, the large surface of the cavernous sinus, which is in close contact with the base of the brain, allows direct cooling of the rostral brain stem. Similar regional SBC has been found in other mammals. In humans, the face and the mucosal surfaces of the nose, which are sources of cool venous blood, are small in relation to the mass of the brain. Moreover, a specialized heat exchanger similar to the carotid rete does not exist in humans, and a substantial fraction of the blood supply to the brain is provided by the vertebral arteries, which have no direct contact with cool venous blood [6]. Cool blood from the skin of the head can flow into the cranium and cool the brain via the emissary veins of the temporal and parietal bones [8]. Moreover, brain cortical arteries can cover distances of 15 to 20 cm in fissures and sulci on the brain surface before reaching their final destinations in the cortex and adjacent white matter [9]. Perforating veins that connect the skin of the head with the venous sinuses in the dura mater allow the venous sinuses to receive cool blood. Thus, the temperature of the blood in the sinuses depends on the relative contributions of extracranial and intracranial inflows. The scalp-sinus pathway may be a source of regional SBC. Another source of regional SBC is the upper respiratory tract. The nasal cavities help to cool arterial blood through heat exchange between inhaled air and blood of the nasal mucosa. The thickness of the bone between the nose and the floor of the anterior cranial fossa permits heat exchange and allows the frontal lobes to be cooled [10]. When these heat exchangers are short-circuited, such as during mechanical ventilation with tracheal intubation, venous blood from the nasal cavities is no longer cooled by ventilation. The high respiratory rate observed in association with body temperature increase most likely functions to increase heat transfer in the nasal cavities, resulting in protection of the brain by decreasing the temperature of the blood supplying the brain.

### 2.2. Thermal Compartments

In humans, two thermal compartments have been described: a central and a peripheral ones [11]. The central compartment includes tissues that are highly perfused under all conditions. Heat exchanges are rapid in this compartment, and, in theory, its temperature is relatively homogeneous. The trunk, head, and also the brain make up the central compartment. The peripheral compartment includes tissues in which the temperature is variable and inhomogeneous (lower limbs, hands, and skin). The temperature in the peripheral compartment is generally 2–4°C lower than in the central compartment and is highly dependent on vascular tonus.

An integrative center that regulates core temperature is located in the hypothalamus [12]. Although the response mechanisms of this center are still not completely known, they are likely to involve neurotransmitters such as norepinephrine, dopamine, acetylcholine, neuropeptides, and prostaglandins such as PGE2. Core temperature undergoes circadian variation that is controlled by the release of melatonin from the suprachiasmatic nucleus. The hypothalamic center also regulates the temperature of the central compartment in response to information from thermoreceptors (monosynaptic pathway), feeding, locomotor activity, or secretion of corticosteroids (plurisynaptic pathway). 

Temperature regulation, or homeothermy, remains a highly active area of research. Two neuronal models of temperature regulation in mammals have been described: the set-point model and the null-zone model. The set-point model includes an adjustable set point and signals from peripheral and/or central temperature-sensitive neurons that are integrated and compared with a set point at the level of the hypothalamus. Thermogenic or thermolytic responses can correct the core temperature toward the set point level [13, 14]. Fever or hypothermia are here considered to result from a shift in the set point [15]. An alternative view is that body core temperature is defended around a “set level” or “null zone” rather than a set point [16]. The existence of this “null zone” has been demonstrated in several species, including humans [16]. The null-zone model is based on the interaction of two variables rather than on the comparison of a variable to a constant set point. Reciprocal cross inhibition between a cold sensor and a heat production effector pathway and a warm sensor and a heat loss effector pathway, with the goal of defending a null zone of core temperature, is the basis of this model [17].

### 2.3. Physiological Fluctuations in Brain Temperature

#### 2.3.1. Brain Activity

Neuronal energy metabolism is primarily used for the restoration of membrane potential after cell depolarization [18]. This suggests a relationship between cellular metabolism and electrical activity. Considering that a large part of the energy used for neuronal metabolism is finally transformed into heat, heat production by the brain is therefore an important characteristic of cerebral metabolic activity. In animals, significant changes of 2 to 3°C in brain temperature have been observed after behavioral stimuli [19, 20]. Increase in intracerebral heat production seems to be the primary cause of the brain hyperthermia observed during behavioral stimuli in animals. Indeed, brain temperature increases first, followed by an increase in blood temperature [21, 22]. In awake subjects (or animals) under these conditions, blood going to the brain is therefore cooler than the brain itself, and the temperature gradient between brain and arterial blood increases with the intensity of behavioral stimuli.

Increased brain activity and metabolism is therefore accompanied by an increase in temperature. Concomitantly, in both animals and humans, there is an increase in cerebral blood flow (CBF). The increase in local cerebral temperature resulting from an increase in local metabolism could be considered one of the causes of local blood flow increase that contributes to the coupling between CBF and metabolism.

#### 2.3.2. General Anesthesia

As previously described, in awake conditions, the brain is warmer than the arterial blood. Depression of cerebral metabolism induced by general anesthesia could affect brain temperature. In rats anesthetized with pentobarbital, urethane, or alpha-chloralose, brain temperature decreases more rapidly than rectal temperature [23]. Under general anesthesia, a healthy brain could therefore be cooler than the blood as was shown in these animal studies.

### 2.4. Where Should We Measure Temperature?

Core temperature can be estimated by measuring the temperature of the lower esophagus, pulmonary artery, nasopharynx, or tympanum [24]. Brain temperature is usually considered a “central” temperature, and in the absence of intracranial pathology, it can be estimated by measuring tympanic or esophageal temperatures. These temperatures are easy to measure and are often used to monitor changes in brain temperature. However, in cases of severe cerebral injury, the estimates yielded by such measurements may be inaccurate [25, 26]. 

In humans, the center of the brain is from 0.5 to 1°C warmer than the epidural space [27]. The brain's surface temperature is always lower than its core temperature, but it is also more variable. For these reasons, it is recommended that temperature sensors are inserted to a depth of at least 1.5 to 2 cm in the brain parenchyma [28]. Several temperature sensors are currently available, all of which use thermocouple technology. Some are designed for intraparenchymal and others for intraventricular use. Analysis of the literature does not allow recommendation of one probe over another. Intraparenchymal probes are the most commonly used [29].

More recently, techniques for the noninvasive measurement of brain temperature with magnetic resonance spectroscopy (MRS) have been developed [30, 31]. Experimental studies in phantoms [31] and experimental models [32] have shown close correlation between temperatures measured by MRS and temperatures measured using implanted probes. MRS has been used to measure temperature in healthy adult human volunteers, during head cooling, in children, in patients with brain tumors, and in patients with ischemic stroke [33].

## 3. Physiological Cerebral Changes Induced by Variations in Brain Temperature

Changes in brain temperature significantly affect vascular, metabolic, and neuronal parameters. Because they have a major impact on cerebral physiology, an understanding of these changes is essential.

### 3.1. Cerebral Metabolism

The relationship between temperature and brain activity has been extensively studied using electrophysiology. Animal studies have shown a close relationship between brain temperature and cerebral metabolic rate of oxygen (CMRO_2_) [34]. Previous studies in rats and dogs reported that temperature changes of more than 1°C significantly altered both functional neurologic outcome and histopathology [35]. Cerebral metabolism changes linearly with brain temperature, with 6 to 8% changes in metabolism per degree Celsius of temperature [36, 37]. In anesthetized dogs at 28°C, cerebral metabolism represents only 50% of that at 37°C [38]. Brain oxygen consumption is therefore dramatically reduced at these temperature levels. It has also been shown that all energy-production pathways in the brain, including the cerebral metabolic rates for glucose (CMR_glu_) and lactate, are reduced by a factor of 2 to 4 with each 10°C decrease in temperature [39]. 

*In vitro*, temperature influences the passive properties of the neuronal membrane and synaptic responses (post-potential). Synaptic transmission is temperature dependent. The effect of temperature on the release of neurotransmitters (excitatory postsynaptic potential) seems more pronounced than the effect of temperature on the synaptic response itself [40, 41]. These temperature-dependent changes in electrophysiological properties can be related to effects on neuronal ion channels. Indeed, some calcium or voltage-gated sodium channels are regulated by temperature [42, 43]. Moreover, glutamate diffusion and toxicity rise in temperature [44]. Temperature changes alter brain neurotransmitter release, reuptake, and diffusion. In animal models of ischemia or focal brain injury, brain temperatures above 39°C are associated with increased levels of extracellular excitatory amino acids, opening of the blood-brain barrier, and an increase in proteolysis of the neuronal cytoskeleton [45]. Excitotoxicity is dependent on brain temperature.

### 3.2. Cerebral Blood Flow

Cerebral blood flow (CBF) also changes with temperature, and these changes are proportional to the changes in cerebral metabolism induced by temperature variations [46]. Due to the physiological coupling between CBF and metabolism, decreased brain temperature induces a concomitant decrease in metabolism and blood flow [47], leading to decreased intracerebral vascular volume and intracranial pressure [48]. However, some studies suggest that the coupling between CMRO_2_ and CBF is nonlinear [49]. During mild hypothermia after cardiac arrest in humans, CBF is low [47]. Rewarming for 24 hours increases CBF to normal values. A recent study of 10 comatose patients who were successfully resuscitated following out-of-hospital cardiac arrest reported an effect of mild therapeutic hypothermia on CBF and cerebral oxygen extraction. The median core temperature at the start of the study was 34.3°C, and this temperature was maintained between 32 and 34°C for 72 hours. The median mean flow velocity in the middle cerebral artery (MFV_MCA_) was low at admission and significantly increased at 72 hours [50]. Median jugular bulb oxygenation (SjbO_2_) was normal in the majority of patients throughout the study. The observation of normal SjbO_2_ together with low MFV_MCA_ strongly suggests that there was decreased cerebral metabolism during the first 24–48 hours of mild therapeutic hypothermia. However, the fact that SjbO_2_ reached a plateau 24–30 hours after admission indicates relatively low cerebral oxygen extraction. These findings suggest that cerebral metabolic coupling may be lost during hypothermia.

### 3.3. Carbon Dioxide, pH, and Oxygen

The level of gaseous carbon dioxide (CO_2_), or CO_2_ partial pressure (PaCO_2_), in arterial blood depends on the solubility coefficient of this gas, which is itself dependent on temperature. As the temperature decreases, the amount of gaseous CO_2_ decreases. In other words, there are fewer bubbles in a champagne bottle when the bottle is cold. Moreover, cellular energetic metabolism, the end products of which are water and CO_2_, decreases with temperature. CO_2_ production is therefore reduced by hypothermia. Thus, for both physical and metabolic reasons, PaCO_2_ decreases with temperature [51]. Similarly, pH is modified by temperature due to changes in PaCO_2_: hyperthermia is accompanied by acidosis, and hypothermia by alkalosis [52]. The CO_2_ gas crosses the blood-brain barrier and transmits the induced modifications (e.g., alkalosis in hypothermia) to the extracellular environment, which regulates the state of arteriolar vascular tone. This explains why hypothermia-induced hypocapnia may cause arteriolar vasoconstriction and a decrease in intracranial pressure [53].

**Figure 2:**
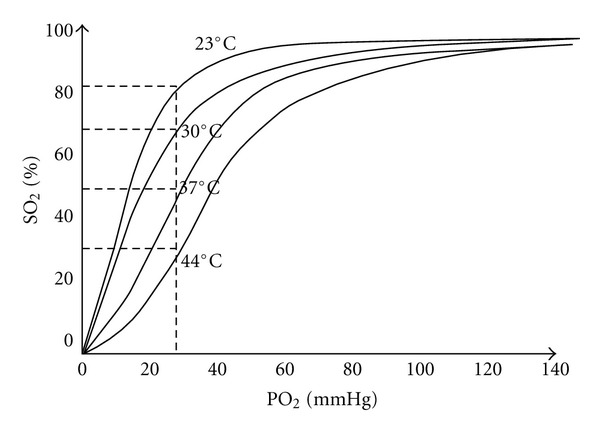
Relationship between oxygen partial pressure (PO_2_) and oxygen saturation of hemoglobin (SO_2_). Hypothermia increases the affinity of hemoglobin for oxygen, according to Tremey and Vigué [51].

The decrease in PaCO_2_ is partly the result of decreased oxygen consumption (O_2_) [53]. This reduction could be beneficial in areas with high ischemic risk. However, the effect is counteracted by an increase in hemoglobin affinity for oxygen that occurs with the decrease in temperature (Figure 2). The increased affinity of hemoglobin for oxygen impedes the diffusion of oxygen to tissues.

### 3.4. Brain Inflammation and Blood-Brain Barrier

In animals, after focal trauma (fluid percussion), the inflammatory response of contused and noncontused brain areas is temperature dependent. Accumulation of leukocytes increases with temperature [54]. These changes in inflammatory processes may play a major role in the posttraumatic cascade. Moreover, the permeability of the blood-brain barrier also seems to depend on brain temperature. An increase in brain temperature can damage the endothelial cells of the brain and spinal cord, leading to diffusion of serum proteins through the blood-brain barrier and contributing to the occurrence of cerebral edema [55]. Even if hyperthermia occurs after a period of four days following trauma (animal model of fluid percussion), brain hyperthermia worsens mortality and increases lesions of the blood-brain barrier and axonal injury [56].

## 4. Changes in Brain Temperature in Neurointensive Care

After major brain injury, brain temperature is often higher than systemic temperature and can vary independently, making the extrapolation of brain temperature from “central” temperature difficult. Rossi et al. [25] found that the number of temperature measurements >38°C in the brain was 15% higher than core body temperature measured simultaneously at the pulmonary artery. The difference between brain and core temperature has been found to be as much as 2°C depending on the characteristics of the patient, probe placement, and interactions with other physiologic variables [25, 57]. As patients become hyperthermic, the difference between brain and core temperature increases, which may indicate that the true incidence of febrile episodes in the brain is even higher than that reported in large observational studies that measured only core body temperature.

### 4.1. Severe Traumatic Brain Injury

Traumatic brain injury (TBI) produces focal or multiple brain injuries, blood-brain barrier disruption, ischemia and reperfusion, diffuse axonal injury and development of cerebral microbleeding, intracranial hematomas, or contusion areas [58]. The primary injury can be followed by secondary injuries that lead to increased cell death and poor neurological outcome [58, 59].

Two studies conducted in sedated patients suffering from severe TBI reported an average brain temperature that was higher by approximately 1°C than the average rectal temperature in the first posttraumatic days [25, 60]. This difference is accentuated when patients become febrile. In the absence of an infectious cause, one explanation of this phenomenon could be a “resetting” of the hypothalamic thermoregulatory center. Autopsies have indeed found a high frequency (42%) of hypothalamic lesions in patients who died after severe TBI [61]. However, other causes could produce an increase in “intracerebral” temperature after TBI. The observed elevation in brain temperature could be related to posttraumatic changes in brain metabolism (hyperglycolysis) [62], in CBF (hyperemia) [63], or in the local inflammatory response (e.g., increased intracerebral interleukin-1*β*) [64]. Decoupling of energy metabolism in cases of brain injury could also contribute to the production of heat; in such cases, ATP synthesis can indeed be short-circuited. The reduction in the proton gradient and the mitochondrial membrane potential accelerates cellular respiration, and respiration is no longer coupled to the phosphorylation of adenosine diphosphate (ADP), becoming a purely thermogenic process (Figure 1).

Inversion of the brain/body temperature gradient, in which the brain temperature falls below the “general” body temperature, is associated with poor neurological prognosis in severe TBI [65]. This phenomenon is also observed during progression to brain death [66]. The decrease in CBF associated with increased intracranial pressure most likely causes a decrease in brain temperature to below the core temperature. Variations in this gradient could therefore reflect the occurrence of cerebral ischemia.

On the other hand, early fever is frequent after TBI and is associated with higher severity at presentation and with the presence of diffuse axonal injury, cerebral edema on the initial head computed tomography scan, systolic hypotension, hyperglycemia, and leukocytosis [2]. Elevations in temperature within the first 24 hours after TBI are attributed to an acute phase response [67]. Other studies have reported that the presence of blood within the cerebrospinal fluid, especially within the intraventricular spaces, may stimulate hypothalamic thermoregulatory centers and lead to increased body temperature [68]. As with all other brain injuries, fever after TBI can be related to the development of infection, to the occurrence of inflammatory responses, and to hypothalamic dysfunction following the injury. Observational studies have found that the occurrence of fever in the first week after injury is associated with increased intracranial pressure, neurologic impairment, and prolonged length of stay in intensive care [69, 70]. Jiang et al. reported a strong relationship between fever and outcome in a study of 846 patients with TBI [71]. Childs et al. suggested that patients who had the highest and lowest average brain temperatures during the first 48 hours after injury were more likely to have a worse outcome and to die [72]. Soukup et al. also reported poor outcome at 3 months in patients with TBI who showed extremes of brain temperature [65]. Recently, Sacho et al. conducted a study in which intraparenchymal brain temperature was measured in severe TBI patients during the first 5 days in the intensive care unit. Brain temperatures within the range of 36.5°C to 38°C during the first 24 hours were associated with a lower probability of death (10–20%). Brain temperature outside this range was associated with a higher probability of death and with poor 3-month neurological outcomes [73]. Evidence for the adverse effects of a small increase in brain temperature on secondary neuronal damage [74] and mortality [4, 56] is now extensive. Hyperthermia causes the release of excitatory amino acids and free radicals, aggravates blood-brain barrier breakdown, amplifies cytoskeletal proteolysis, and increases cerebral metabolic rate [75–77]. Recently, Stocchetti et al. described impact of pyrexia on neurochemistry and cerebral oxygenation after acute brain injury in humans [78]. During the onset of fever, cerebral oxygenation was preserved, and no signs of anaerobic metabolism (stable concentrations of glucose, lactate, pyruvate and glutamate, and lactate to pyruvate ratio) were recorded, possibly because of a concomitant increase in CBF.

Therapeutic cooling or targeted temperature management has been proposed as a neuroprotective treatment for TBI. From a historical perspective, Fay first introduced neurological therapeutic hypothermia in 1943 in a case of severe TBI [79]. The primary neuroprotective benefit of therapeutic hypothermia has been attributed to reduction of CMRO_2_, which is strongly linked to oxygen and glucose consumption and lactate production in neurons [80, 81]. However, many neuroprotective effects of hypothermia have been described, including reduced metabolism (permitting a decrease in interstitial lactate accumulation and the maintenance of physiological tissue pH balance) [82], reduced intracranial pressure (ICP) [83], stabilized blood-brain barrier, reduced free radical production, decreased accumulation of lactic acid and other neurotoxins, enhanced glucose utilization, facilitaed antiinflammatory responses and anti-apoptotic pathways, and reduced release of excitotoxic neurotransmitters such as glutamate [82, 84–87]. The intracranial pressure decrease induced by hypothermia occurs through multiple mechanisms: decrease in CMRO_2_ and thus in CBF and cerebral blood volume, decrease in ischemic edema, and decrease in PaCO_2_. 

A number of studies with animal models have shown that hypothermia can improve outcome after experimental TBI [84, 88, 89]. These results have led to clinical trials. Studies including patients with refractory raised ICP showed a decrease in ICP during cooling [84, 90–93]. One prospective multicentric randomized study did not find any beneficial effect on outcome [48]. However, in a subgroup of patients who were hypothermic on admission, 52% of those assigned to the hypothermia group had poor outcomes, while 76% of those assigned to the normothermia group had poor outcomes. A recent meta-analysis suggests that treatment with hypothermia may decrease mortality and improve neurologic outcome if treatment is maintained more than 48 hours [94]. Guidelines for the management of severe TBI have limited prophylactic hypothermia recommendations to level III because of potential confounding factors [95]. 

Therapeutic hypothermia appears to be an attractive tool, but its handling requires experienced teams. In our neurointensive care unit, we recommend its use in severe TBI patients presenting hypothermia on arrival at the hospital and as a third-line option for the treatment of raised intracranial pressure (target temperature 33°C for at least 48 hours).

Fever can also be regarded as an adaptive response that enhances the ability to control infection. Induction of normothermia may impair this adaptive response. In fact, the use of antipyretics has been reported to prolong the evolution of certain types of bacterial and viral infections [96, 97]. Studies have shown a correlation between febrile response and increased survival rate in patients with community-acquired pneumonia, *Escherichia coli, Streptococcus pneumonia,* and *Pseudomonas aeruginosa* sepsis [98–101]. Fever also has the direct effect of inhibiting the replication of some microorganisms, and it enhances the antibacterial effect of a variety of antibiotics [102, 103]. Schulman et al. reported higher mortality rates in critically ill patients with aggressive treatment (treatment when temperature was >38.5°C) compared to a permissive group (treatment when temperature was >40°C) [104]. Recently, however, Schortgen et al. described the effect of external cooling for fever control during septic shock in a multicenter-randomized controlled trial. Body temperature was lower in the cooling group after 2 hours (36.8°C *versus* 38.4°C), resulting in a significant decrease in vasopressor dosage and better shock reversal. Moreover, day 14 mortality rate was better in the cooling group (19% *versus* 34%) [105]. Therefore, in this study, fever control during septic shock was demonstrated to be safe. However, several important points of this study should be emphasized. First, the main source of infection was the lung and not the abdomen; in cases involving the latter, deleterious effects of fever control have been shown in experimental models [106, 107]. Second, most of the patients in Schortgen's study have received appropriate antimicrobial therapy, thereby mitigating the potential negative effect of fever control on host defenses [102]. Further, it is important to emphasize that the goal in this study was fever control and not induction of hypothermia. Of note, in several previous studies, an increased risk of acquisition of infection after mild therapeutic hypothermia was demonstrated [108, 109].

### 4.2. Severe Subarachnoid Hemorrhage

Nontraumatic subarachnoid hemorrhage (SAH) primarily occurs due to intracranial aneurysm rupture [110]. Sudden internal bleeding causes high ICP. Bleeding in subarachnoid spaces, sometimes with intraventricular hemorrhage or intraparenchymal hematoma, follows rupture of an aneurysm. Brain tissue hypoxia can occur in relation to significant CBF decrease and edema formation [111]. After a severe SAH, brain temperature is usually higher than core temperature [112]. An attractive hypothesis involves the potential role of the degradation products of heme. The heme molecule is degraded by heme oxygenase to biliverdin, iron, and carbon monoxide (CO) [113]. In rats, intraventricular injection of CO increases body temperature by more than 1°C [114]. 

A prospective study in patients admitted for severe SAH found a relationship between brain temperature and survival [112]. In TBI, when the measured brain temperature is lower than the body temperature (bladder), the prognosis is very poor. This temperature decrease could also be related to a significant decrease in CBF.

In the acute phase of SAH, alterations in body temperature regulation are common. Fever, defined as body temperature >38.3°C, occurs in up to 72% of aneurysmal SAH patients [115, 116]. Noninfectious fever, usually beginning in the first 3 days, is common in patients with SAH [117]. In patients with intraventricular hemorrhage, body temperature is persistently increased (plateau) instead of presenting spikes [68]. Refractory fever during the first 10 days after SAH is associated with increased mortality, severe functional disability, and cognitive impairment among survivors [3]. Cumulative fever burden, defined as the sum of time at body temperature >38.3°C in the first 13 days, is associated with worse outcome and with later and often incomplete recovery in good-grade patients and potential late recovery in poor-grade patients [118]. Moreover, fever induces cerebral metabolic distress, and elevated lactate/pyruvate ratios have been documented using microdialysis during febrile episodes. In acohort study, Oddo et al. found an association between fever and cerebral metabolic distress and showed that cerebral metabolic distress can be reduced with fever control independently of intracranial pressure management [119]. Induced normothermia was related to significant reduction in the lactate/pyruvate ratio and fewer episodes of cerebral metabolic crisis, supporting the view that fever control may be “neuroprotective.” This evidence suggests that fever could be detrimental and that its control could reduce metabolic distress.

A recent review describes fever incidence, impact, and treatment in patients with SAH [120]. In SAH, fever is associated with worse outcome and increased length of stay [121] and has detrimental effects independent of vasospasm. Fever has also been linked to symptomatic vasospasm independent of hemorrhage severity or the presence of infection [113, 122]. This association could be due to inflammatory activation after SAH [123], which might be implicated in the development of both phenomena. In addition to disease severity and to the amount of blood in the subarachnoid space, the presence of intraventricular hemorrhage is a strong risk factor for fever development [3, 68]. Fever exacerbates ischemic injury [75], worsens cerebral edema, increases intracranial pressure [25], and may lead to a decreased level of consciousness. 

Hypothermia has not been studied in severe SAH patients being treated in intensive care units. Deep intraoperative hypothermia has been proposed to protect brain tissue from surgery-related ischemic damage. A recent review by the Cochrane collaboration evaluated the effect of intraoperative mild hypothermia on postoperative death and neurological deficits in patients with intracranial aneurysms [124]. The authors concluded that there were insufficient data to draw any conclusions and that therapeutic hypothermia should therefore not be recommended during surgery in patients with poor-grade aneurysmal SAH. Recently, guidelines for the management of aneurysmal SAH have proposed recommendations on anesthetic management during surgical and endovascular treatment. Induced hypothermia during aneurysm surgery is not routinely recommended but may be a reasonable option in selected cases (Class III, level of evidence B) [125]. The IHAST study compared 499 patients randomly assigned to an intraoperative hypothermia group during surgery for intracranial aneurysm (target temperature 33°C) *versus* 501 patients in a normothermia group (36.5°C) [126]. The aim of the study was to determine whether intraoperative cooling during open craniotomy resulted in improved outcome among patients with acute aneurysmal SAH. The results did not show any significant differences between the two groups. Other studies have not shown any benefit of hypothermia on cognitive function or neuropsychological outcome after SAH [127, 128].

Therapeutic hypothermia is not routinely used or recommended in severe SAH. In practice, we do not use intraoperative cooling because of lack of evidence for its use. 

### 4.3. Stroke

Ischemic stroke is one of the major causes of adult disability in industrialized countries [129]. Stroke causes permanent brain damage and long-term impairment. In the central core regions of the insult, neuronal cells undergo death within minutes. Surrounding this core, CBF levels may fall below functional thresholds but above the threshold for cell death; this area has been called the penumbra [130]. The penumbral zone permits cell survival only for a period of time, but at least some of the tissue in this zone is potentially salvageable.

After ischemic stroke, the temperature in the areas of the brain affected by ischemia is higher than the temperature in the unaffected parts of the brain and the rest of the body [33]. Clinical trials of therapeutic hypothermia in patients with ischemic stroke have been conducted based on observations that in animal models hypothermia reduces the size of cerebral infarcts by more than half [131]. Furthermore, in stroke patients, higher body temperature is associated with poorer outcome [4].

The processes that determine brain temperature after human ischemic stroke are not fully understood. There may be dissociation between metabolic activity and heat generation in ischemic brain. A systemic response to the increase in systemic inflammatory cytokines after stroke could also increase brain temperature. Interleukin-6 (IL-6) triggers the release of other proinflammatory cytokines, and its presence is important for the generation of fever [132]. Higher levels of IL-6 and acute phase proteins are associated with poorer functional outcome after stroke [133, 134], and one potential mechanism for the association with poor outcome is an increase in brain temperature. Whiteley et al. recently studied 44 patients with acute ischemic stroke and found an association between levels of IL-6, as well as downstream acute-phase proteins such as C-reactive protein and fibrinogen, and changes in brain or body temperatures over the first 5 days after stroke [135]. In this study, brain temperature was recorded at hospital admission and 5 days after stroke using multivoxel magnetic resonance spectroscopic imaging of normal-appearing brain and of the acute ischemic lesion, which was defined by diffusion-weighted imaging [35]. The mean temperature in DWI-ischemic brain soon after admission was 38.4°C (95% confidence interval (CI) 38.2–38.6), while in DWI-normal brain the mean temperature was 37.7°C (95% CI 37.6–37.7). The mean body temperature was 36.6°C (95% CI 36.3–37.0). Higher levels of interleukin-6, C-reactive protein, and fibrinogen were associated with higher temperature in DWI-normal brain at admission and at 5 days.

Therapeutic hypothermia has been proposed as a neuroprotective strategy after ischemic stroke. In patients suffering from cerebral ischemia, therapeutic hypothermia may minimize the extent of injury by modulating various steps of the ischemic cascade [136]. Target temperature management reduces neuronal excitotoxicity by blocking glutamate and dopamine release, leading to reduced calcium influx and lipid peroxidation and thus attenuating free radical production [85]. Temperature-related reduction of free radical production has been associated with decreased neuronal damage during both the ischemic and reperfusion phases [137]. Another hypothesis is that therapeutic hypothermia may favor the upregulation of stress response genes that produce antiapoptotic proteins. These gene products are translocated into the nuclei, where they regulate gene expression favoring cell survival [138, 139].

In experimental stroke studies, mild hypothermia (32–34°C) seemed to be superior to other temperatures tested; for example, it resulted in a larger reduction in infarct volume than 27°C [140] and better tolerance than 30°C [141]. A number of studies suggest that hypothermia is neuroprotective when applied early after the stroke, and that it remains beneficial if the duration of cooling is prolonged [142–144]. It should be noted that in many animal studies therapeutic hypothermia is initiated before or at the onset of ischemic stroke, whereas in clinical situations, patients typically reach the hospital several hours after the onset of the injury. Furthermore, most patients receive hypothermia for several days, whereas animal models use hypothermia only for short cooling periods. The rewarming phase after therapeutic hypothermia is also crucial because rapid rewarming may enhance deleterious ischemic effects. Berger et al. have shown that slow rewarming significantly reduces the infarct volume compared to fast rewarming [145]. 

A recent review found 17 relevant clinical studies of the use of hypothermia after ischemic stroke (4 observational studies, 5 self-controlled clinical trials, and 8 parallel-controlled clinical trials) [129]. The observational studies show that admission temperature is a prognostic factor for poor neurological outcome and mortality in ischemic stroke [146–148]. The self-controlled studies suffer from lack of a proper control group, and their results are not sufficiently robust to justify the conclusion that hypothermia influences stroke outcome [149–153]. Of the parallel-controlled clinical trials that have been conducted to date, only one showed improvement in NIHSS (National Institutes of Health Stroke Scale) and significant differences in mortality rate with hypothermia and craniectomy combination compared to craniectomy alone [154]. Two randomized double blind studies have been completed. One did not report any difference between hypothermia and normothermia for mortality or NIHSS at 24 hours or 72 hours in patients undergoing craniectomy [155]. Mortality has been found to be similar between hypothermia and control groups in all randomized blinded clinical trials [155, 156]. 

The literature suffers from lack of evidence supporting the use of mild therapeutic hypothermia on ischemic stroke patients.

## 5. Conclusion

After severe brain injury, brain temperature is usually not measured, although several studies have shown that it may differ significantly from core temperature. Measurement of body temperature often underestimates brain temperature, especially in situations in which the central nervous system is vulnerable. Dissociation between brain and body temperature could be a sign of poor prognosis. After major brain injury, brain temperature, similarly to intracranial pressure, should be continuously monitored using *in situ* measurement; such measurement should most likely be a part of the multimodal monitoring of patients to prevent secondary injury to the brain.

Fever management should take into consideration the protection of the brain from secondary insults as well as the capacity to fight against infections. Fever should most likely be treated aggressively in the first days of TBI, SAH, or stroke, but randomized controlled trials are needed to assess the risk-benefit ratio. Therapeutic hypothermia has yielded promising results in animal models of TBI, SAH, or stroke, but its usefulness in clinical practice is still debated. In severe TBI, therapeutic hypothermia permits control of intracranial pressure elevation, but its effects on outcome and mortality have not been conclusively demonstrated. In patients with poor-grade aneurysmal SAH, therapeutic hypothermia is not recommended during aneurysmal surgery. The benefit of hypothermia in reducing infarct size in humans after ischemic stroke is not clear.
